# Impact of medication reviews on drug‐related problems (DRPs) in older patients living in nursing homes in West Occitania

**DOI:** 10.1111/fcp.12820

**Published:** 2022-08-11

**Authors:** Alice Zacarin, Cyrielle Gonzales, Delphine Nigon, Antoine Piau, Haleh Bagheri

**Affiliations:** ^1^ Department of Medical and Clinical Pharmacology, PharmacoVigilance, PharmacoEpidemiology and Drug Informations, Faculty of Medicine, INSERM UMR 1027 University Hospital and Faculty of Medicine Toulouse France

**Keywords:** Elderly, Drug Related Problems, Medication Review, Nursing Home

## Abstract

Despite several guidelines for preventing potentially inappropriate medication (PIM) use in older, their prescription rates remain high (25%). The aim of this study was to determine the impact of medication reviews (MRs) on the drug‐related problems (DRPs) in older patients in Elderly Residential Care Homes (nursing homes [NHs]). DRP was defined as an event or circumstance involving drug therapy that actually or potentially interferes with desired health outcomes. We conducted a retrospective study on 2819 residents of the 46 NHs between 1 January 2017 and 31 December 2018. Drug prescription was analysed according to European EU(7)‐PIM list and START/STOPP list. We then linked each PIM to an appropriate type of DRP. Three months later, we requested the ‘updated’ drug prescriptions to assess whether the recommendations had been followed. A total of 17 850 prescription lines were registered. A DRP was identified for 25% of them. Following the second request, 13 NHs (28%) responded. About 26% (*n =* 1188) of the overall prescriptions lines identified as a DRP involved these 13 NHs, which resulted in a recommendation being made during the first MR. Data from the second MR suggested that 53.9% (*n =* 640) of recommendations were followed with the requested change: 32.0% involved drug withdrawal (*n =* 381), 9.7% concerned dose adjustment (*n =* 115) and 6.5% required drug changes (*n =* 77). Our results show the benefit impact of MR on the quality of drug prescription in older NH residents. MRs should be one of the tools used to improve drug prescriptions in the elderly.

## 1 INTRODUCTION

In 2020, 20.5% of the French population were over 65 years of age [1]. Approximately 10% of subjects >75 years of age live in residential care homes for the elderly (nursing homes [NHs]), and this figure rises to 30% for those >90 years of age [2]. Occitania is the second largest region in France with an older than average population compared with the rest of France. The ageing index (ratio of the number of inhabitants aged 65 or over per 100 young people under the age of 20) is the fourth highest in France: 89.1 versus a national average of 72.2 [3]. Because of multiple comorbidities, older patients require multiple medication and are therefore at risk of potentially inappropriate medication (PIM) use, with increased hospital admissions and mortality due to adverse drug reactions (ADRs), although the latter are potentially preventable [4, 5, 6]. PIMs are one cause of drug‐related problems (DRPs) defined as ‘an event or circumstance involving drug therapy that actually or potentially interferes with desired health outcomes’ [5]. Our previous study carried out among older elderly adults in NHs suggested that 77% of this population had at least one PIM [7]. Medication reviews (MRs) are one way of preventing PIM use and ensuing DRPs in older adults. MRs offer a structured and systematic approach to individual drug therapy analysis in order to suggest improvements in medication management and potentially reduce iatrogenic events and their consequences [8]. Some studies have highlighted the impact of MR on PIM use in older individuals. Lenander et al. suggested a decrease in the combined use of three or more psychotropic drugs (one of the PIM indicators according to Swedish guidelines) post‐MR [9]. Another study assessed post‐MR changes in PIM use in geriatric care units between 2007 and 2013 and demonstrated a significant improvement in five of the six selected quality indicators [10].

The PAAPI programme (Optimising Inappropriate Prescriptions in the Older people) was set up by the Toulouse Regional Pharmacovigilance Centre (CRPV), during the second half of 2016 in an attempt to promote the rational use of prescribed drugs in institutionalised older subjects in eight departments in West Occitania (Ariège, Aveyron, Haute‐Garonne, Gers, Lot, Hautes‐Pyrénées, Tarn and Tarn et Garonne). At the end of 2016, a letter outlining the project and signed by the Regional Healthy Agency (Agence Régionale de Santé, ARS) and the Pharmacovigilance Centre was sent to the NHs in West Occitania (*n =* 353). From 2017 onwards, we conducted an on‐site visit to each participating NH to explain the project in more detail [7].

The main aim of our study is to determine the impact of MRs on PIM use and then on DRP assessed by the follow‐up of the recommendations according to the type of DRP before and after MRs in older patients living in NHs participating in the PAAPI.

## 2 METHOD

### 2.1 Study setting and population

After the first mailshot to NHs in the region of West Occitania, a total of 46 NHs volunteered to participate in the study (13%) including a total of 2819 residents. An on‐site visit was then organised to meet the coordinating physician and explain the project. The NHs enrolled in the study were located in different departments of West Occitania: Ariège: 3 (14%), Aveyron: 3 (6%), Haute‐Garonne: 22 (18%), Gers: 1 (4%), Hautes‐Pyrénées: 5 (17%), Lot: 4 (14%), Tarn: 3 (5%) and Tarn et Garonne: 5 (22%). We conducted a retrospective study on the 2819 residents of the 46 NHs participating in the PAAPI programme between 1 January 2017 and 31 December 2018. The coordinating physician in each NH then forwarded anonymous data to us regarding the residents' medical data and drug prescriptions with the consent of their respective general physician (GP). Data were collected in a single random review day.

Among the 46 NHs (with 2819 residents) participating in the first MR, 13 NHs (28%) with 959 residents (34%) responded to the second MR request and sent us their residents' updated prescription data.

We excluded residents who died or were receiving palliative care during the study period.

### 2.2 Data collection

**FIGURE 1:**
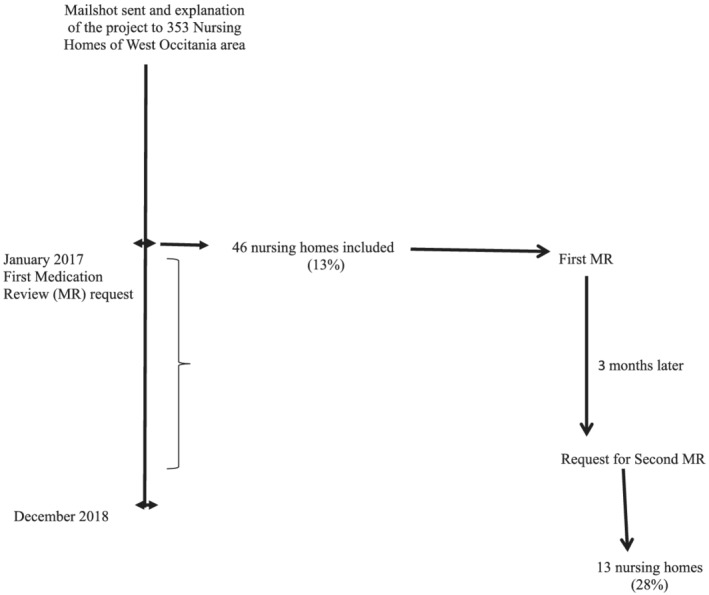
Flowchart of PAAPI setting up

The following sociodemographic and clinical data were collected for each resident: age, gender, medical history and comorbidities and iso‐resource group (IRG) and drug prescriptions. The IRG measures an older individual's degree of autonomy on a scale of 1 to 6: IRG 1 corresponds to total dependency, long‐term prescriptions and their dosages. Drug prescriptions were reviewed on three occasions in the Pharmacovigilance Centre: by the PAAPI assistant who recorded anonymous data in an Excel database and then transmitted the data to a resident pharmacist for the first analysis and MR and finally to a senior pharmacologist for final MR and review of the previous analysis. For each PIM identified, a comment was added to emphasise the potential risk labelled as ‘Risk identification’ along with a suggestion to improve the drug prescription labelled as ‘Recommendation’. This drug prescription analysis corresponded to the ‘First MR’ which was returned to the coordinating physician for subsequent transmission to the GPs. Three months later, we contacted the coordinating physician again to request the ‘updated’ drug prescriptions for residents for whom at least one recommendation was sent to assess whether the recommendations had been followed. This second drug prescription analysis corresponded to the ‘Second MR’ (Figure 1). Eye drops and drugs indicated for acute conditions (antibiotic therapies, parenteral anticoagulants, etc.) were excluded from the MR.

### 2.3 Medications review—PIM and DRP

Various criteria have been used to identify and limit PIM use, including measures based on clinical judgement regarding individual patients (implicit criteria) and criterion‐based measures (explicit criteria) [10, 11]. During the MRs, we used the criterion‐based European EU(7)‐PIM list to identify PIM (medications with an unfavourable risk/benefit ratio when a safer alternative is available or when their efficacy has not been proven in a given indication) [12]. However, we also adopted an approach by not classifying as inappropriate several prescriptions in the following cases:

- Serious conditions (epilepsy, Parkinson's disease, heart failure, diabetes, etc.) where the patient's complete medical record is required in order to recommend appropriate prescription changes;
- When the EU(7) PIM list does not suggest an alternative (e.g., amiodarone);
- When the patient's status (age and comorbidities) does not permit certain additional diagnostic investigations to justify a prescription (proton pump inhibitor in elderly patients with anaemia, for instance).

Moreover, since the EU(7)‐PIM list mainly allows detection of overuse or misuse, we also used a STOPP/START classification system to identify underuse in various cases [13]. All PIMs were also assessed according to the Anatomical Therapeutic Chemical (ATC) classification [14].

Then, we linked each PIM or case of underuse of drug to an appropriate type of DRP according to Cipolle et al. This task was facilitated because of the way in which our NHs were organised [15]. Indeed, there is no patient compliance issue in these institutions. DRPs identified for each drug line prescription were then classed according to five categories:

- Additional drug requirement
- Unnecessary drug
- PIM
- Dose too high or too low
- Potential ADRs

Based on the potential DRPs identified following the MRs, we put forward recommendations which were also defined on the basis of the following five categories [8]:

- Initiating drug—example: antiplatelet drugs when indicated;
- Drug withdrawal—example: benzodiazepines with a prolonged half‐life;
- Warning or evaluation of benefit/risk ratio—example: reminder to assess renal function when a potentially nephrotoxic drug has been prescribed; memantine in patients with advanced dementia;
- Dose adjustment—example: excessively high dosage of Z drugs;
- Drug replacement—example: citalopram replaced by other antidepressant drugs.

Each line of prescription generating an identified DRP and could lead to one or more recommendations. For example: PIM could generate both warning and drug withdrawal.

### 2.4 Statistical analysis

We carried out a descriptive analysis of the patients' sociodemographic and clinical data and of the characteristics of the NH and drugs prescribed for NH residents. Descriptive analyses were performed using number and percentage for qualitative variables and mean ± standard deviation (SD), minimum (Min) and maximum (Max) for quantitative variables. Qualitative and quantitative variables were compared using chi‐square and *t*‐student tests, respectively. The characteristics of drugs (Table 3) were compared after the first and second MRs using paired tests (paired *t*‐test for comparing means and Mac Nemar test for frequencies).

Finally, in order to assess the type of recommendations more followed by NHs after the first MR, we compared for each drug the rate of prescription after the first and second MRs using the symmetry Mc Nemar test. A 0.05 level of significance was applied, and statistical analyses were conducted using R software.

## 3 RESULTS

### 3.1 General population and NH data

Table 1 summarises the residents' characteristics during the first and second MRs. There is no significant difference between the characteristics of residents included in the first and second MRs, except for the arterial hypertension and cardiovascular diseases more frequent in the population of the second MR. Table 2 shows the characteristics of NHs participating only in the first MR and those participating in both MRs. The presence of an in‐house pharmacy and a coordinating physician was associated with participation in the second MR. No difference was found according to the status of NH or the number of GP consulting in the NH.

**TABLE 1:** Characteristics of all residents of nursing home included and the comparison of their characteristics between those participating or not to the follow‐up

Characteristics of residents	All residents	Residents participating to follow‐up	Residents not participating to follow‐up	*p*
Number of residents	2819	959	1860	‐
Age, mean (± SD)	89 (±8)	88 (±9)	89 (±7)	0.550
Gender (%)				
Female	2072 (74%)	709 (74%)	1363 (73%)	0.710
Male	747 (26%)	250 (26%)	497 (27%)	
GIR (%)				
1–2	1012 (36%)	359 (37%)	653 (35%)	0.430
3–4	629 (22%)	206 (21%)	423 (23%)	
5–6	93 (3%)	35 (4%)	58 (3%)	
No information	1085(38%)	359 (37%)	726 (39%)	
Medical histories				
Arterial hypertension	1198(42%)	328 (34%)	870 (47%)	<0.001
Cardiovascular diseases	686(24%)	199 (21%)	487 (26%)	0.001
Metabolic diseases	669(24%)	225 (24%)	444 (24%)	0.800
Dementia	726(26%)	240 (25%)	486 (26%)	0.520
Osteoarticular diseases	444(16%)	140 (15%)	304 (16%)	0.220
Inflammatory bowl	314(11%)	110 (12%)	204 (11%)	0.690

**TABLE 2:** Characteristics of nursing homes participating in the first or both medication review (MR)

Characteristics of nursing homes	All NH	Including in the first MR	Including in the first and second MR	*P*‐value (chi‐square test)
Number of nursing homes status (%)	46 (100%)	33	13	
Public	25 (54%)	16 (48%)	9 (69%)	
Private	21 (46%)	17 (51%)	4 (31%)	
In‐house pharmacy (%)				0.030
Yes	17 (37%)	9 (27%)	8 (62%)	
No	29 (63%)	24 (73%)	5 (38%)	
General practitioner (GP)				
0–5	14 (30%)	9 (27%)	5 (38%)	
6–15	19 (41%)	15 (45%)	4 (31%)	
>15	13 (28%)	9 (27%)	4 (31%)	
Nursing home coordinator GP				0.050
Yes	25 (54%)	15 (45%)	10 (77%)	
No	21 (46%)	18 (45%)	3 (23%)	

Abbreviation: NH, nursing home.

### 3.2 Identification of DRP

Following our initial request and the collection of data relating to 46 NHs, a total of 17 850 prescription lines were registered for 2819 residents. As explained in Section 2, DRP could not be identified for 359 lines of prescription, concerning amiodarone (*n =* 119) and PPI (*n =* 240).

A DRP was identified for 4524 prescription lines (25%) classed as follows: wrong drug (inappropriate drug) in 45% (*n =* 2042), unnecessary drug prescription in 31% (*n =* 1416), ADR in 13% (*n =* 595), excessively high dose in 8% (*n =* 367) and the need for additional therapy in 2% (*n =* 104) of prescriptions.

Each category of DRP could lead to different types of recommendation. Recommendations were as follows: drug withdrawal in 39% of cases (*n =* 1777), drug change in 24% (*n =* 1078), reduced dose in 18% (*n =* 832), warning in 16% (*n =* 749) and drug initiation in 2% of cases (*n =* 88).

**FIGURE 2:**
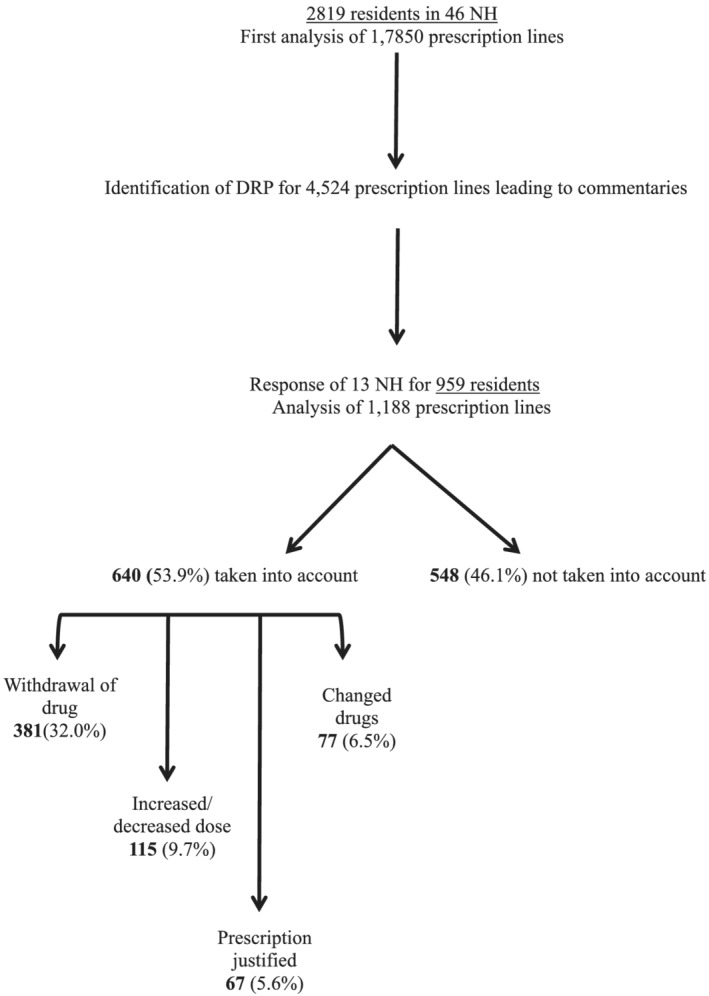
Flow chart for identification of drug‐related‐problem and analysis of the follow‐up of recommendations by nursing home (NH)

Following the second request, 13 NHs (28%) responded and sent us their residents' drug prescription data (959 residents). About 26% (*n =* 1188) of the overall prescriptions lines identified as a DRP involved these 13 NHs, which resulted in a recommendation being made during the first MR. Data from the second MR suggested that 53.9% (*n =* 640) of recommendations were followed with the requested change: 32.0% involved drug withdrawal (*n =* 381), 9.7% concerned dose adjustment (*n =* 115) and 6.5% required drug changes (*n =* 77). Moreover, in 67 cases (5.6%) involving the ‘Warning or evaluation of benefit/risk’ recommendation, the NH in question responded and argued the validity of their prescription and noncompliance with the recommendations. In 46.1% of cases, the NHs did not follow the recommendation (Figure 2).

Table 3 shows medication characteristics for residents in the 13 NHs participating in both MRs (*n =* 959). The number of residents with 0–4 drugs increased during the second MR, but those with >9 drugs did not decrease significantly. Furthermore, the number of residents with at least one DRP decreased significantly during the second MR (44%, *n =* 421) compared with the first MR (61%, *n* = 589).

**TABLE 3:** Characteristics of drugs of residents of nursing home (*n =* 959) participating to the first and second medication review (MR)

Medications	First MR	Second MR	*P*‐value
Number of residents	959	959	
Mean of drugs (± SD; MIN‐MAX)	6.3 (±3.2; 0–19)	6 (±3; 0–18)	0.008^a^
Number of drugs			
0–4	295 (31%)	337 (35%)	0.040^b^
5–9	509 (53%)	494 (51%)	
>9	155 (16%)	128 (13%)	
Residents with at least one DRP	589 (61%)	421 (44%)	0.0001^b^

Abbreviation: DRP, drug‐related problem; MR, medication review.

^a^ Paired *t*‐test.

^b^ Mc Nemar test.

The DRP and rate of compliance with the recommendations were also analysed for each drug classed according to the ATC (Table 4). The follow‐up of recommendations was significative for the majority of drugs except fibrate, urapidil, diclofenac, nicorandil, bromazepam and lorazepam due to the sample size.

**TABLE 4:** Main categories of ATC, drug‐related problems, recommendations and the rate of modifications done after medication reviews in 959 residents

Anatomical therapeutic chemical classification	Drugs	*n* of drugs	Drug‐related problems	Recommendations	*n* of modification
N05 psycholeptics					
N05A antipsychotics					
N05AA06	Cyamemazine	23	Wrong drug	Withdrawal of drug therapy/change of drug therapy	7 (30%)
N05AD01	Haloperidol	39	Wrong drug	Withdrawal of drug therapy/change of drug therapy	15 (38%)
N05BA12	Alprazolam	42	Wrong drug	Withdrawal of drug therapy/change of drug therapy	18 (43%)
N05BA08	Bromazepam	7	Wrong drug	Withdrawal of drug therapy/change of drug therapy	4 (57%)
N05BA06	Lorazepam	11	Wrong drug	Withdrawal of drug therapy/change of drug therapy	3 (27%)
N05BB01	Hydroxyzine	13	Wrong drug	Withdrawal of drug therapy	13 (100%)
N05C hypnotics and sedatives					
N05CD06	Lormetazepam	19	Dose too high	Decreased dose	7 (37%)
N05CF01	Zopiclone	76	Dose too high	Decreased dose	32 (42%)
N05CF02	Zolpidem	51	Dose too high	Decreased dose	11 (22%)
N06 psychoanaleptics					
N06A antidepressants					
N06AB04/N06AB10	Citalopram/escitalopram	87	Wrong drug	Change of drug therapy	42 (48%)
N06AB05	Paroxetine	51	Wrong drug	Withdrawal of drug therapy/change of drug therapy	11 (22%)
N06AA09	Amitriptyline	13	Wrong drug	Withdrawal of drug therapy	6 (46%)
A Alimentary tract and metabolism					
A02B Drugs for peptic ulcer and gastro‐oesophageal reflux disease					
A02BC01‐A02BC05	Proton pump inhibitors	283	Unnecessary drug therapy	Withdrawal of drug therapy/decreased dose	79 (28%)
A03F propulsives					
A03FA01	Metoclopramide	18	Wrong drug	Withdrawal of drug therapy	9 (50%)
C cardiovascular system					
C10A lipid modifying agents					
C10AA05	Statin	115	Unnecessary drug therapy	Withdrawal of drug therapy	32 (28%)
C10AB	Fibrate	5	Wrong drug	Withdrawal of drug therapy	3 (60%)
C08C selective calcium channel blockers with mainly vascular effects					
C08CA04	Nicardipine	37	Wrong drug	Withdrawal of drug therapy	9 (24%)
Vasodilators					
C01DX16	Nicorandil	6	Wrong drug	Withdrawal of drug therapy	2 (33%)
C02C antiadrenergic agents, peripherally acting					
C02CA06	Urapidil	13	Wrong drug	Withdrawal of drug therapy	4 (31%)
M01A anti‐inflammatory and antirheumatic products, nonsteroid					
M01AB05	Diclofenac	8	Wrong drug	Withdrawal of drug therapy	4 (50%)
M02AA17	Niflumic acid	2	Wrong drug	Withdrawal of drug therapy	2 (100%)
M02AA13	Ibuprofen	4	Wrong drug	Withdrawal of drug therapy	2 (50%)

Abbreviation: ATC, Anatomical Therapeutic Chemical classification.

## 4 DISCUSSION

We conducted this study in order to illustrate the benefit of drug prescription MRs for the older residents of NHs and to estimate the rate of compliance with our recommendations. According to our descriptive analysis, about one fourth of drug prescription lines was classed as PIM. Cool et al. performed a retrospective study in 175 NHs of West Occitania in 229 residents and found a rate of exposure to PIM in 71% of them according to Laroche list [16, 17]. Nyborg et al. reported at least one PIM in 43% of cases in older individuals residing in NHs based on the NORGEP‐NH criteria in Norway [18]. Other authors found a higher prevalence (82.6%) in Brazilian NHs according to Beers' criteria [19]. Data variability could be due to the different criteria and PIM list used as the reference.

Our results suggest improved drug prescription for both qualitative and quantitative criteria with a significant decrease of the number of patients exposed to at least one PIM during the second MR and fewer patients exposed to 5–9 or >9 drugs although the mean overall number of drugs did not significantly decrease. Recently, the study of Debacq et al. performed in 125 patients admitted in a geriatric unit showed a low PIM prescription rate at 6 months after patients discharge [20]. In France, Cool et al. also suggested a reduction of PIM prescribing in NH residents after a geriatric intervention [21]. Leguelinel‐Blache suggested that the number of patients taking at least one PIM decreased from 30.6% before to 6.1% after MR associated with a mean saving of €232 per patient [22]. Moreover, Choukroun et al. found the benefit impact of MR on DRP in older outpatients with cancer [23].

As regards the recommendation compliance rate, our data suggest that almost 54% of NHs complied. Our recommendations were primarily followed for drugs classified as PIMs. This is corroborated by sound evidence such as the withdrawal of benzodiazepines with a prolonged half‐life (alprazolam), a reduction in the dosage of Z drugs for elderly adults or the replacement of citalopram or paroxetine, which are not recommended as first‐line antidepressants in this population. Despite the difficulty of deprescribing proton pump inhibitors (PPI) due to symptomatic rebound secretion following prolonged exposure [24, 25], recommendations to reduce PPI doses were also followed. In the case of primary prevention statins, our data also demonstrate significant compliance. However, recent data suggest some benefits of statins in primary prevention in patients presenting several risk factors but more clarification is required in order to establish which older individuals could benefit from primary prevention statins based on broader considerations of risk and function. The risks of negative functional and cognitive outcomes must also be weighed against the potential of prolonged (and possibly even improved) life expectancy. Current international cardiovascular disease prevention guidelines provide little specific guidance for doctors considering statin withdrawal in older adults in the context of declining health status and short life expectancy [26]. Our study data suggest significant withdrawal of primary prevention statins which are deemed unnecessary. Last but not the least, other well‐followed recommendations concerned nicardipine and urapidil, which are classed as PIMs according to the EU(7)‐PIM list. Similar results showing the beneficial impact of MRs on prescription quality have been suggested in other studies [9, 10]. To our knowledge, this is the first French study to evaluate the positive impact of MR in NH, including almost 3000 residents who could be deemed representative of the older population in our region and France based on demographic data [2, 3].

Among the NHs participating in the first MR, few of them (26%) responded to the second MR. Several factors could be used to determine NH participation in the MR. Our results suggest that the presence of an in‐house pharmacy and a coordinating physician could prompt participation. Indeed, the mailing of medical and medication records is an onerous task and health professionals do not always have time for this in addition to their usual workload.

This study is limited by the lack of data on the potential ADRs and the outcome for residents for whom recommendations were followed compared with those who continued to take drug(s) identified as a potential risk. Our sample size did not facilitate statistical comparison. The low reply of NHs for the follow‐up of drug prescriptions also remains a limitation of this study and the difficulty to set up this intervention. This point remains crucial in order to estimate the real impact of different actions on the morbidity and mortality of older residents. The beneficial impact of deprescribing and/or intervention remains controversial according to the literature. Kua et al [27] suggested a significant reduction in terms of deprescribing on PIM use (59%) and all‐cause mortality (26%) in a subgroup of residents, while other studies reported the insignificant impact of deprescribing on the clinical outcome for older residents given the high risk of bias in the studies under consideration [28]. De Souto Barreto et al. also demonstrated the lack of impact of reduced benzodiazepine use in NHs due to external factors [29]. Finally, another potential limitation of a MR process remains the operational feasibility in long term because it requires some resources. This process should be supported by sanitary authorities and in Occitania; the examples of PAAPI and OPTIMAGE that begun in 2006 and 2017, respectively, show the interest of these actions [30].

In conclusion, our results show the benefit impact of MR on the quality of drug prescription in older NH residents. NH participation largely depends on the structure of each institution. MRs should be one of the tools used to improve drug prescriptions in the elderly. In order to estimate the impact of MR on the morbidity and mortality of older individuals, further study should be performed to compare the outcome of two categories of residents with or without recommendations follow‐up.

## CONFLICT OF INTEREST

The athors have no conflicts of interest that are directly relevant to the content of this article.

## AUTHOR CONTRIBUTIONS

A Zacarin and C Gonzales collected the data and contributed to the data analysis. D Nigon did statistical analysis. A Piau reviewed the paper and advised for the method of study. H Bagheri coordinated all the study.

## ETHICS STATEMENT

Not applicable.

## INFORMED CONSENT

Not applicable.

## Data Availability

Not applicable.
